# Zinc selenide (ZnSe) nanoparticle coated with green seaweed (***Ulva fasciata***) hydroalcoholic extract as an anti-leishmanial compound on ***Leishmania major***

**DOI:** 10.1371/journal.pone.0321219

**Published:** 2025-04-29

**Authors:** Zahra Atef, Fatemeh Livani, Faramarz Koohsar, Roghiyeh Faridnia, Ganesh Yadagiri, Hamed Kalani

**Affiliations:** 1 Infectious Diseases Research Center, Golestan University of Medical Sciences, Gorgan, Iran; 2 Clinical Research Development Unit (CRDU), Sayad Shirazi Hospital, Golestan University of Medical Sciences, Gorgan, Iran; 3 Laboratory Sciences Research Center, Golestan University of Medical Sciences, Gorgan, Iran; 4 Department of Veterinary Biosciences, The Ohio State University, Columbus, OH, USA; Mohanlal Sukhadia University, INDIA

## Abstract

This study focused on evaluating the effectiveness of zinc selenide nanoparticles coated with green seaweed (*Ulva fasciata*) (ZnSe-*Uf*) against *Leishmania major* (*L*. *major*) in light of increasing drug resistance in the treatment of cutaneous leishmaniasis and the growing necessity for new therapeutic options. Key characteristics of ZnSe-*Uf*, including shape, size, functional groups, zeta potential, and polydispersity index, were analyzed in detail. The study investigated the effects of different concentrations of ZnSe-*Uf* compared to meglumine antimoniate (MA; used as the control), on both the promastigote and amastigote forms of *L*. *major*, calculating the selectivity index (SI) for each. Analysis revealed that the dominant functional group in ZnSe-*Uf* was C-H stretching, attributed to polysaccharides, lipids, and proteins. The size of ZnSe-*Uf* ranged from 228.2–242.8 nm (P = 0.37), with a polydispersity index of 0.31–0.33 (P = 0.85), and a zeta potential ranging from -35.6 mV to -57.9 mV (P = 0.07) over a period of 90 days. The lethal concentration 50 (LC_50_) for ZnSe-*Uf* was 7.61 μg/mL, while for MA it was 17.37 μg/mL on promastigote (P = 0.03). On amastigote, the LC_50_ was 24.3 μg/mL for ZnSe-*Uf* and 12.3 μg/mL for MA (P = 0.04). The SI was 27.55 for ZnSe-*Uf* and 41.26 for MA (P = 0.02). The lower LC_50_ for MA on amastigote indicated its better effectiveness on *L*. *major* compared to ZnSe-*Uf*, suggesting that ZnSe-*Uf* may have a lower ability to concentrate in macrophages compared to MA. However, ZnSe-*Uf* still showed anti-leishmanial activity and was non-toxic to macrophages (SI > 10), indicating the need for further investigation on animal models.

## Introduction

Cutaneous leishmaniasis (CL) is a major infectious disease worldwide, caused by the intracellular protozoan *Leishmania*, primarily *Leishmania major* (*L*. *major*) and *L*. *tropica* [1]. The primary clinical manifestation of CL is the development of skin lesions following the bite of an infected female mosquito belonging to the *Phlebotomus* or *Lutzomyia* genus [2]. Globally, approximately 0.7–1.3 million new cases are reported annually, with 80% of these occurring in just ten countries. However, it is widely believed that the true prevalence of CL is significantly exceeds the reported cases, potentially being 6–10 times higher [3].

While CL skin lesions may heal on their own within a year, timely treatment is crucial for several reasons. Treatment not only helps prevent secondary infections and restricts the spread of the disease, but it also minimizes scarring [1]. There are various treatment options available for CL, including both medicinal and non-medicinal treatment options available for CL [4]. Pentavalent antimonials have been the primary treatment for CL for over a century. However, the emergence of drug resistance against these antimonials has become a significant concern, leading to the need for alternative treatment regimens when the initial treatment fails [1,5]. Antimony-resistant *Leishmania* parasites often exhibit reduced drug uptake due to the downregulation of the aquaglyceroporin 1 transporter. Additionally, overexpression of ATP-binding cassette (ABC) transporters facilitates the efflux of thiol-metal complexes, thereby reducing drug accumulation within the parasites [6,7]. Moreover, the elevated intracellular levels of thiols, such as glutathione and trypanothione, can form complexes with antimony, sequestering it away from its target sites and mitigating its effects [7,8]. Genetic alterations, including intrachromosomal amplification of genes like MRPA (a type of ABC transporter), contribute to resistance by enhancing the efflux of drugs. Mutations and changes in gene expression can also lead to a decreased sensitivity to antimonials [6,9]. Furthermore, antimony induces oxidative stress within *Leishmania* cells. Resistant strains have adapted by optimizing their antioxidant responses, allowing them to survive despite the presence of the drug [6].

The effectiveness of a drug depends on its ability to accumulate at the targeted organ with a sufficient concentration. Failure to achieve this can result in ineffective treatment [10]. Inadequate drug accumulation at a specific site is primarily due to limitations in the drug delivery system [11]. Enhancing the drug delivery system, along with the antimicrobial properties of various nanoparticles, has been found to reduce drug dosage and improve effectiveness. This can minimize drug side effects and the development of drug resistance [12,13]. The impact of different nanoparticles, either alone or in combination with other substances, has been investigated in the context of CL [12]. For examples, gold nanoparticles have shown immunomodulatory effects and can inhibit thioredoxin reductase, contributing to their anti-leishmanial activity [14,15]. Additionally, biodegradable polymeric nanoparticles allow for controlled drug release, enhancing the therapeutic index of existing drugs such as amphotericin B (AmB) and meglumine antimoniate [15]. Research has also shown that liposomes and niosomes can improve the bioavailability of hydrophilic drugs like paromomycin, which may struggle to penetrate skin barriers [16].

Among all the different types of nanoparticles, zinc selenide (ZnSe) stands out as a multifunctional one, formed by the combination of zinc and selenium [17–19]. Recent research has compared the antibacterial properties of zinc oxide and selenium oxide nanoparticles, and the results suggest that while ZnO NPs reveal strong antibacterial activity, selenium nanoparticles indicated antioxidant properties. This specifies a potential synergistic effect when combining these two types of nanoparticles for enhanced antimicrobial applications [20]. However, there is currently a lack of data regarding the effects of ZnSe nanoparticles on parasites, particularly *L*. *major*. Therefore, our focus will be on exploring the potential of ZnSe nanoparticles in this context.

The use of herbal drugs for treating CL lesions has a long history, with the first detailed description of such lesions dating back to almost 1,000 AD [21]. It is worth noting that a significant proportion of important drugs in modern medicine are derived from plants, with paclitaxel being a notable example [22,23]. Numerous studies have demonstrated the effects of herbal drugs on *Leishmania* species [24]. Green seaweed (*Ulva fasciata*) is abundant in bioactive compounds with various medicinal properties, including antioxidant, antibacterial, and anti-inflammatory effects. These compounds can enhance the therapeutic efficacy of the synthesized nanoparticles [25,26]. However, it is unknown whether green seaweed has any effects on the *Leishmania* parasite. Therefore, further investigation is warranted to explore the potential impact of green seaweed on *Leishmania* parasites.

Therefore, the aim of this study is to investigate in vitro anti-leishmanial effects of ZnSe NPs coated with green seaweed (*Ulva fasciata*) extract on *L. major*.

## Materials and methods

### Ethical considerations

This study was conducted following the approval of the Ethics Committee of Golestan University of Medical Sciences, under ethical code: IR.GOUMS.REC.1399.258.

### Preparation of *Ulva fasciata* hydroalcoholic extract

*Ulva fasciata* was collected from the shores of the Persian Gulf and confirmed by a botanist (registry code: 12775-6). It was then washed with distilled water, dried, and then powdered. The maceration method was used to extract compounds, where 100 g of powdered *Ulva fasciata* was mixed with 400 mL of 80% methanol in distilled water (v/v) [22] in a closed container in the dark. The mixture was stirred twice a day for 3 days. Afterwards, the solution was centrifuged at 10,000 ×g for 15 min and then passed through a 0.8 µm pore size syringe filter with a polyethersulfone membrane (Corning Inc., NY, USA), and then through a 0.2 µm pore size syringe filter with a polyethersulfone membrane (Corning Inc.). The resulting solution was concentrated on a rotary evaporator under vacuum conditions at 45 °C until 10 mL remained. The sample was then turned into powder using a lyophilizer and stored at 4 °C until use.

### ZnSe NPs preparation

Zinc selenide nanoparticles (ZnSe NPs) were synthesized using sodium selenite (Na_2_SeO_3_) as the source of selenium ions (Se) and zinc nitrate (Zn(NO_3_)_2_) as the source of zinc ions (Zn). The method for preparing ZnSe NPs was previously described in detail by Daun and Yao [27]. Briefly, a Teflon autoclave containing 20 mL of 1M NaOH solution was supplemented with 0.298 g of Zn(NO_3_)_2_ and 0.173 g of Na_2_SeO_3_. The mixture was subjected to supersonic vibration, and then 10 mL of 80% (v/v) hydrazine hydrate solution was gradually added while stirring. Subsequently, 10 mL of deionized water was added and the solution was stirred for 10 minutes. The autoclave was then heated to 180 °C and maintained at this temperature for 4 hours. After the reaction, the solution was centrifuged at 20,000 ×g for 15 minutes. The resulting sediment was collected, washed three times with absolute ethanol and distilled water, and then freeze-dried at room temperature. The resulting powder was stored at 4 °C until further use.

### ZnSe NPs coated with *Ulva fasciata* extract (ZnSe-*Uf*)

All steps are the same as those explained for the preparation of ZnSe NPs, with the only difference being the use of 0.471 g of the prepared *Ulva fasciata* extract powder in 10 mL of deionized water instead of just deionized water. The optimization of the ratio of nanoparticles to *Ulva fasciata* extract was achieved by conducting the experiments with varying variable values of pH [28,29], temperature [30], and reaction time [31], as well as different concentrations of the extract while keeping the against a constant concentration of ZnSe NPs [30]. The results were determined by using UV-Vis spectroscopy method [32]. As mentioned above, washing steps were performed on the resulting sediment and finally the solution turned into powder and was kept at 4 °C until use.

### ZnSe-*Uf* morphology

The structure of ZnSe-*Uf* was analyzed using field emission scanning electron microscopy (FESEM). A small amount of ZnSe-*Uf* powder was mixed with a drop of absolute ethanol, placed on a conductive substrate, dried, and then studied.

### ZnSe-*Uf* functional groups

Fourier-transform infrared spectroscopy (FTIR) procedure was applied using the Nicolet IR100 FTIR spectrometer (Madison, WI, USA) to examine the chemical bonds and functional groups in ZnSe-*Uf* in comparison with ZnSe NPs. The samples were prepared with double distilled water, sonicated for 5 min in a water bath sonicator, and examined in the wavenumber range of 400–4000 cm^−1^, with 50 scans and a resolution of 4 cm^−1^.

### ZnSe-*Uf* zeta potential, polydispersity index (PDI), and size

The dynamic light scattering (DLS) procedure was applied using the HORIBA SZ-100 nanoparticle analyzer (Horiba Scientific SZ-100, Kyoto, Japan) at 25 °C and a scattering angle of 90 degrees to determine the zeta potential, PDI, and size of ZnSe-*Uf*. The samples were diluted, prepared with double distilled water, and examined on day 0 and 90 to measure their stability.

### Parasite

The *L. major* of MRHO/IR/75/ER strain was studied in this investigation. The parasite was kept in the laboratory by being passed through in phenol red-free RPMI 1640 medium (Gibco Laboratories, Grand Island, NY, USA) with the addition of 10% fetal bovine serum (FBS) (Gibco) and antibiotics (Gibco), as described by Mirzaei et al. [22].

### ZnSe-*Uf* effects on promastigotes

ZnSe-*Uf*, ZnSe NPs, meglumine antimoniate (MA) (used as the control), and *Ulva fasciata* extract were dissolved in DMSO to their respective saturation points. The concentrations of each compound after dissolution were documented. To determine the lethal concentration 50 (LC_50_), 40 concentrations of each compound were prepared in phenol red-free RPMI 1640 in 2-fold serial dilutions. These concentrations were then evaluated on promastigotes in 96-well microplates to identify the range that induced death rates from 10% to 90%. When necessary, adjustments were made to refine the range of concentrations to achieve the desired death spectrum. Subsequently, 150 µL of each prepared concentration was added in triplicate to the wells, along with 150 µL of medium containing 10,000 stationary-phase promastigotes. The DMSO concentration in each well was maintained below 0.2%. Three wells with promastigote-containing medium were considered as negative control to which no drug was added. The plates were then incubated at 24 °C for 72 hours. The death rate of the promastigotes was assessed using the MTT Cell Proliferation Assay Kit (Abcam, Cambridge, MA, USA), according to the manufacturer’s instructions with slight modifications. After the 72-hour incubation, the microplates were centrifuged, and the supernatant medium was carefully removed. To the precipitated promastigotes, 50 µL of MTT reagent and 50 µL of medium were added. Three separate wells, each containing 50 µL of MTT reagent and 50 µL of medium, were prepared to serve as background controls for optical density (OD) correction. After 3-hour incubation at 37 °C, 150 µL of MTT solvent was introduced into each well. The microplates were gently shaken for 15 min, then centrifuged. The supernatant from all wells was carefully transferred to a new microplate, centrifuged once more, and analyzed using a microplate reader (Bio-Rad, CA, USA) at a wavelength of 590 nm. All OD measurements were adjusted by subtracting the background values, and the promastigote death rate was determined using the following formula:


$$Deathrate=\frac{\text{OD control}-\text{OD sample}}{\text{OD control}}\times 100$$

### ZnSe-*Uf* effects on amastigotes

The J774A macrophage cell line was employed in this study. A total of 5,000 J774A cells, suspended in 200 µL of medium, were seeded into each well of the microplates, which were then incubated at 37 °C for 24 hours. Following incubation, the medium in each well was removed, and 50,000 stationary-phase promastigotes in 300 µL of fresh medium were introduced. The microplates underwent a subsequent incubation at 37 °C for another 24 hours, after which the medium was discarded. Each well now contained amastigote-infected macrophages, with three wells designated as the negative control groups. Ten concentrations below and ten above the calculated LC_50_ for promastigotes, prepared in 2-fold serial dilutions, were added to the wells in triplicate in 150 µL of the same medium, bringing the final volume of each well to 300 µL. The microplates were incubated at 37 °C for 72 hours. The rate of amastigote death was determined using the MTT Cell Proliferation Assay Kit, following the same protocol previously described for promastigotes.

### ZnSe-*Uf* effects on J774A cell line and selectivity index (SI)

A total of 10,000 J774A cells per well were seeded in 150 µL of RPMI 1640 medium and incubated at 37 °C for 24 hours. Subsequently, 40 concentrations of the aforementioned compounds in 150 µL of the same medium were added. The rate of cell death was assessed using the MTT Cell Proliferation Assay Kit, as previously described. The SI for each compound was calculated using the following formula. A compound was considered as safe if its SI was greater than or equal to 10 (SI ≥ 10):


$$Selectivityindex=\frac{\text{Cell line}\text{LC}50}{\text{Amastigote LC}50}$$

### Data analysis

The lethal concentration 50 (LC_50_) values for promastigote, amastigote, and the J774A cell line was calculated using GraphPad Prism v.8 software (San Diego, CA, USA). Group comparisons were subsequently analyzed with IBM SPSS v.20 software (Armonk, NY, USA).

## Results

### ZnSe-*Uf* morphology

**Fig 1:**
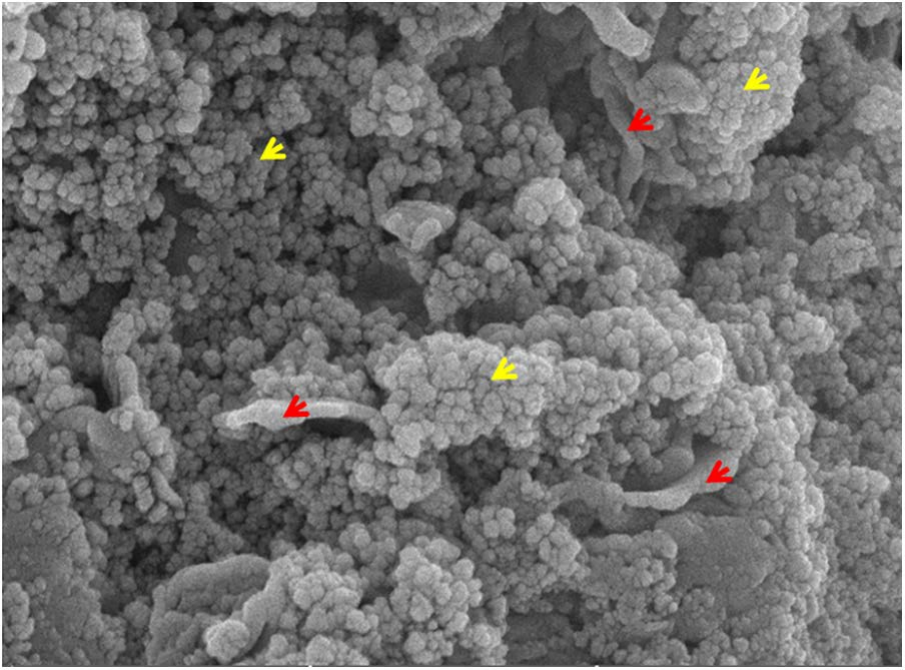
Zinc selenide nanoparticles coated with green seaweed (*Ulva fasciata*) extract. Red arrow head shows *Ulva fasciata* extract and yellow arrow head shows Zn selenide nanoparticles.

FESEM was employed to verify the attachment of *Ulva fasciata* extract to ZnSe NPs. Fig 1 displays the FESEM image of ZnSe-*Uf*. The ZnSe NPs appear nearly spherical, while the extract is recognizable as plate-like structures. The observations suggest a strong interaction between the extract and the nanoparticles.

### ZnSe-*Uf* functional groups

**Fig 2:**
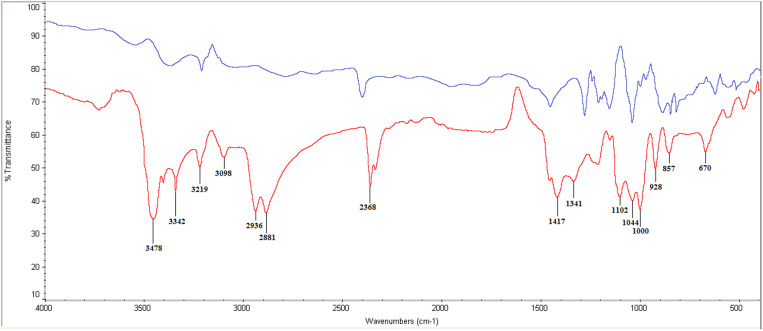
FTIR diagram for zinc selenide nanoparticles coated with green seaweed (*Ulva fasciata*) extract (red line) and zinc selenide nanoparticles alone (blue line).

Using the FTIR procedure, the functional groups in ZnSe-*Uf* were identified and compared to those in ZnSe NPs. The peak with a shoulder at 3478 cm^-1^ in ZnSe-*Uf*, which is attributed to the amino group (NH_2_) [33], and the sharp bond at 3342 cm^-1^, which is characteristic vibration of hydroxyl group (OH) [34], are associated with *Ulva fasciata* extract. The stretching bond at 3219 cm^-1^ is attributed to hydroxyl group (OH) vibration [35]. Peaks observed at 3098 cm^-1^, 2936 cm^-1^, and 2881 cm^-1^ are linked to C-H stretching vibrations found in polysaccharides, lipids, and proteins [36–39], all of which originate from *Ulva fasciata* extract. The absorption spectrum featuring a shoulder at 2368 cm^-1^ is attributed to the amine group (N-H) and also sourced from *Ulva fasciata* extract. The symmetric bond at 1417 cm^-1^ is attributed to carbonyl group (C=O) stretching [40]. A weak peak at 1341 cm^-1^ is corresponded to phosphorus (P=O) stretching [41]. Furthermore, the absorptions at 1102 cm^-1^ and 1044 cm^-1^ are attributed to C-O vibrations [42]. The absorption bond within the range of 928–670 cm^-1^ are linked to C-H stretching vibrations of aromatic compounds [43] (Fig 2).

### ZnSe-*Uf* zeta potential, polydispersity index (PDI), and size

The stability of ZnSe-*Uf* stability was assessed by measuring zeta potential, PDI, and size on days 0 and 90. Statistically, no significant changes were observed in zeta potential (P = 0.53), PDI (P = 0.28), or size (P = 0.75) of ZnSe-*Uf* over this period. The properties of ZnSe-*Uf* are presented in Table 1.

**Table 1:** Size, polydispersity index, and zeta potential of nanomedicine.

	Time^a^ (days)	Size (nm) ± SD	Polydispersity index ± SD	Zeta potential (mV) ± SD
**Nanodrug**	0	228.2 ± 18.32	0.31 ± 0.05	‐35.6 ± 2.78
	90	242.8 **±** 11.07	0.33 ± 0.07	‐57.9 ± 3.15
**P value**		0.37	0.85	0.07

^a^The nanodrug kept at 25 °C for 90 day.

### ZnSe-*Uf* effects on promastigotes

**Fig 3:**
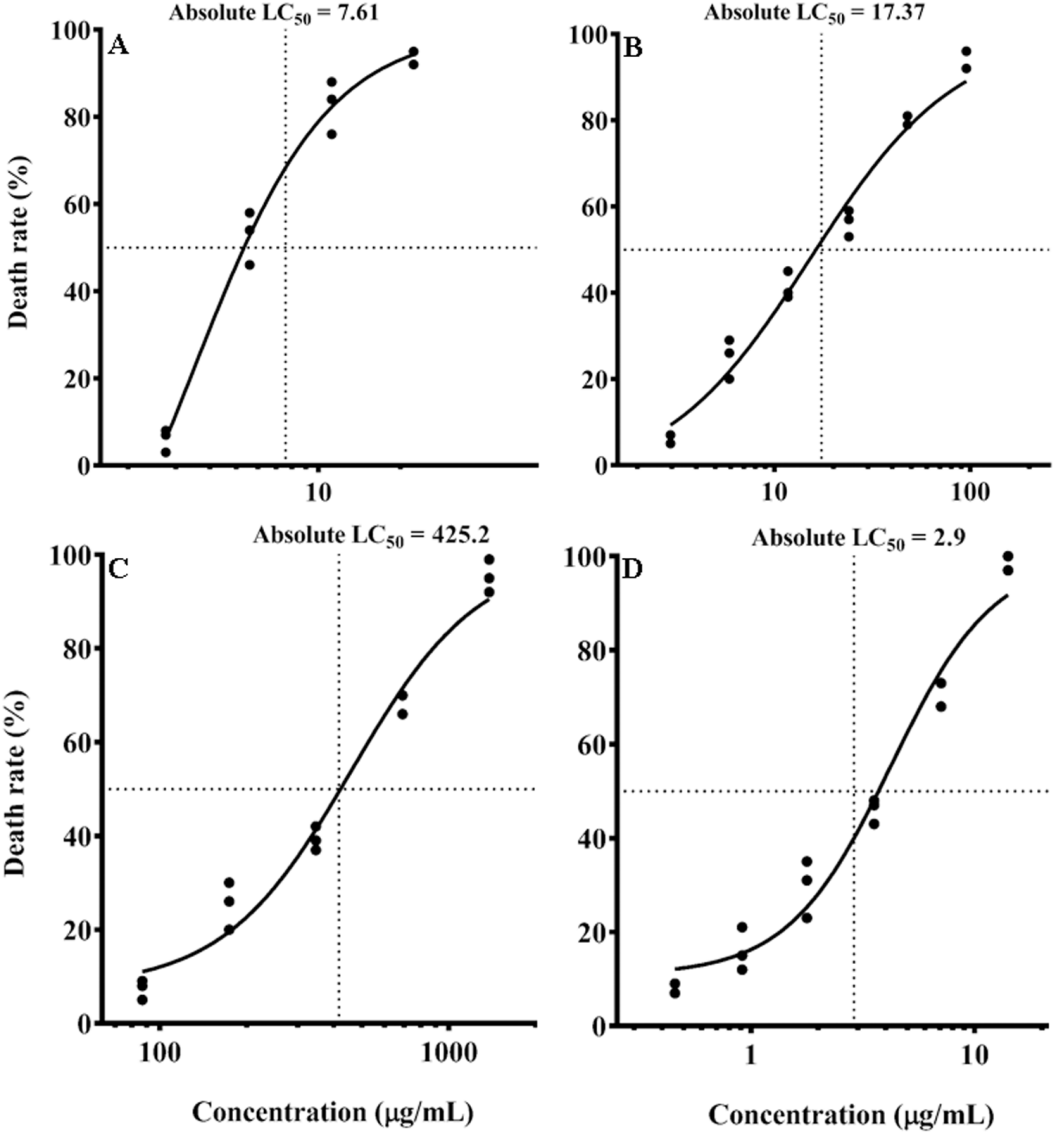
Different compounds LC_50_ for promastigote. (A) Zinc selenide nanoparticles coated with green seaweed (*Ulva fasciata*) extract (2.75–22.03 μg/mL); (B) Meglumine antimoniate (2.95–94.43 μg/mL); (C) *Ulva fasciata* extract (87.09–1393.54 μg/mL); (D) zinc selenide nanoparticles (0.45–14.62 μg/mL); LC_50_ = Half maximal lethal concentration.

The effective concentration range was determined to be 2.75–22.03 μg/mL for ZnSe-*Uf* and 2.95–94.43 μg/mL for MA. The LC_50_ values were calculated as 7.61 μg/mL for ZnSe-*Uf* and 17.37 μg/mL for MA (P = 0.03). Details regarding the effective concentration ranges and LC_50_ of other compounds studied are provided in Fig 3.

### ZnSe-*Uf* effects on amastigotes

**Fig 4:**
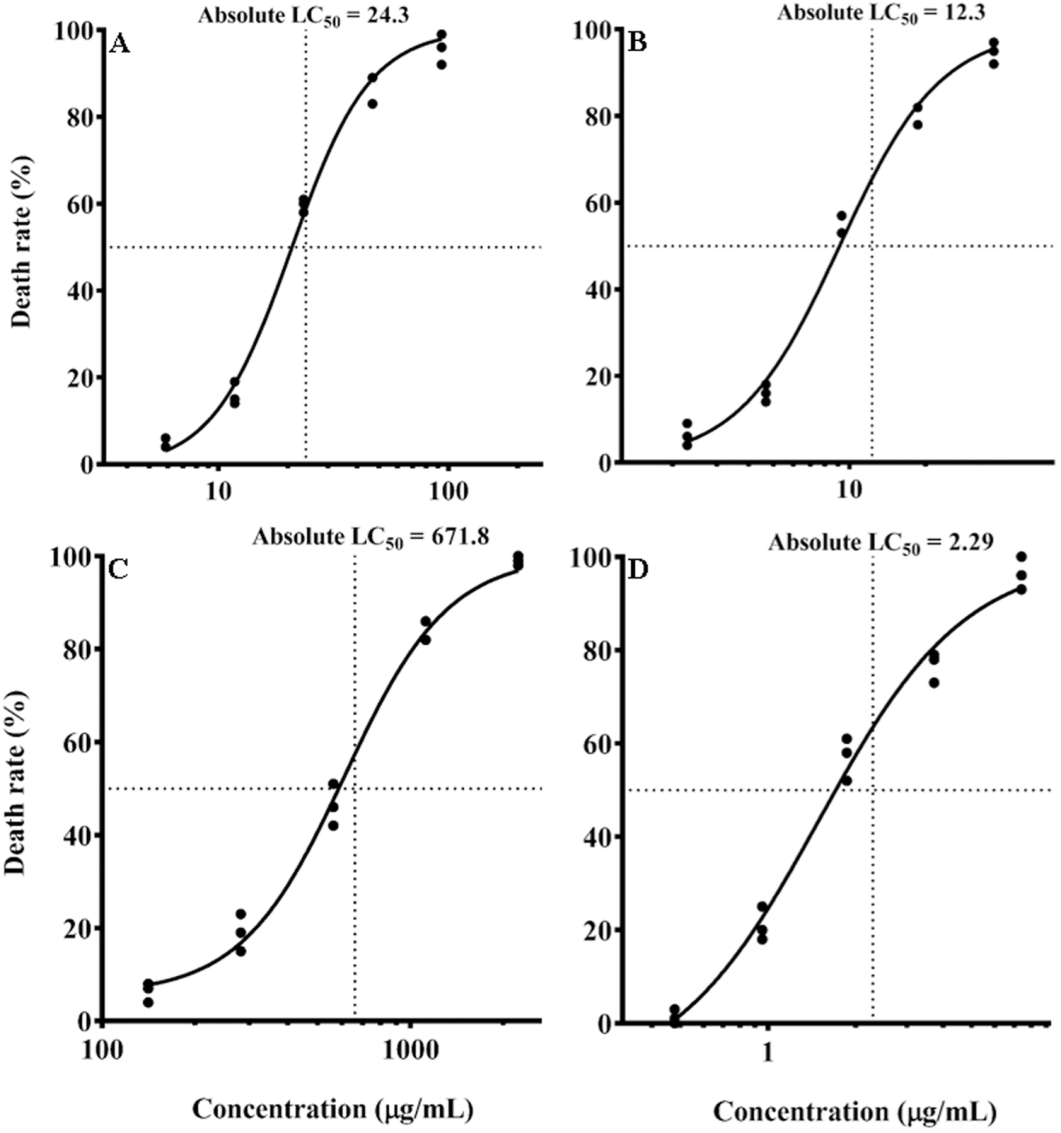
Different compounds LC_50_ for amastigote. (A) Zinc selenide nanoparticles coated with green seaweed (*Ulva fasciata*) extract (5.9–94.4 μg/mL); (B) Meglumine antimoniate (2.34–37.44 μg/mL); (C) *Ulva fasciata* extract (142.5–2280 μg/mL); (D) Zinc selenide nanoparticles (0.47–7.52 μg/mL); LC_50_ = Half maximal lethal concentration.

The effective concentration range for ZnSe-*Uf* was 5.9–94.4 μg/mL, while for MA, it ranged from 2.34–18.72 μg/mL. The LC_50_ value for ZnSe-*Uf* was calculated as 24.3 μg/mL, compared to 12.3 μg/mL for MA. The difference in LC_50_ values between ZnSe-*Uf* and MA was statistically significant (P = 0.04). Details on the effective concentration ranges and LC_50_ values of other investigated compounds are presented in Fig 4.

### ZnSe-*Uf* selectivity index (SI)

To determine the safety of each compound, the SI values were calculated. The SI was found to be 27.55 for ZnSe-*Uf* and 41.26 for MA (P = 0.02). It was measured 127.4 for *Ulva fasciata* extract and 3.88 for ZnSe NPs.

## Discussion

The treatment of CL has consistently encountered numerous challenges, with drug resistance being a significant obstacle that highlights the need for the discovery of new therapeutic options. Researchers have shown growing interest in utilizing NPs to enhance drug delivery systems, reduce the required doses and side effects of existing medications, and improve their overall efficacy [44]. Currently, the only nano-based drug approved by the US Food and Drug Administration (FDA) for CL treatment is liposomal amphotericin B, AmBisome^®^. However, its administration is limited to intravenous infusion, as it proves ineffective when delivered intradermally or subcutaneously. This limitation significantly restricts its applicability in CL treatment [45].

In our study, the size of ZnSe-*Uf* on day 90 was measured at 242.8 nm. While some research suggests that the size and shape of NPs significantly influence their ability to penetrate the skin, other studies have disputed this claim [46–48]. Research indicates that smaller NPs (5–10 nm) can penetrate skin more effectively than larger ones, primarily due to their ability to navigate through the skin’s lipid layers and intercellular spaces. However, particles smaller than 10 nm may be rapidly cleared from the body through renal excretion [49,50]. Hair follicles provide a significant route for NP penetration. Studies suggest that NPs sized between 7–20 nm can access these follicles effectively, which aids their overall absorption into deeper skin layers [51,52]. NPs in the range of 20–100 nm have been shown to penetrate skin more efficiently compared to either smaller or larger particles. For instance, polystyrene NPs around 20–40 nm can distribute well within the skin, while larger particles (100–200 nm) exhibit restricted penetration [49,51]. Therefore, the size of ZnSe-*Uf* (242.8 nm) can be one of its limitations, which requires an in vivo study on the lesion caused experimentally by *L*. *major* in a mouse model.

The shape of nanoparticles, whether spherical, rod-shaped, or triangular, affects their interaction with skin tissues. For example, studies have demonstrated that different shapes of silver nanoparticles (AgNPs) exhibit varying degrees of skin penetration capabilities. Rod-shaped AgNPs showed higher penetration rates compared to spherical or triangular counterparts [52]. In this research, as illustrated in Fig 1, the NPs exhibit a spherical morphology, while the extracts display a plate-like structure that appears to be aggregated. This unique composition set is apart from typical nanoparticles and necessitates additional study on a mouse model.

It is important to recognize that, alongside the characteristics of nanoparticles themselves, various skin-related factors may influence their ability to permeate the skin barrier. Hydration plays a vital role in enhancing the permeability of the skin barrier. Studies indicate that increased skin hydration can improve the transdermal delivery of both hydrophilic and hydrophobic compounds, which is essential for effective NP penetration [53]. Lower pH levels may enhance the release of certain NPs from their carriers due to changes in solubility or degradation rates. Additionally, variations in pH can influence the ionic interactions between NPs and skin components, potentially facilitating or impeding penetration [54]. Studies have shown that NPs penetrate more readily through compromised skin barriers compared to intact skin. This is particularly relevant for therapeutic applications where damaged skin may be more susceptible to NP delivery. The presence of lesions or abrasions increases the surface area available for NP entry and may disrupt the lipid matrix of the subcutaneous, allowing easier access for NPs [54,55].

Measuring the zeta potential of NPs is crucial for assessing their tendency to accumulate in the body and understanding their electrostatic properties [56]. Anionic nanoparticles bind to positively charged sites on skin cells primarily through electrostatic interactions and specific receptor-mediated mechanisms involving integrins and proteoglycans [57–59]. The zeta potential of ZnSe-*Uf* measured in our study was -35.6 mV on day 0 and -57.9 mV on day 90 (P > 0.05), suggesting its stability throughout the investigation period. The zeta potential between +10 and -10 indicates a tendency of NPs to accumulate, which is considered a disadvantage. In contrast, values exceeding +30 or below -30 are one of the strengths of NPs, indicating their unwillingness to accumulate [56]. Based on the findings, ZnSe-*Uf* exhibits a highly anionic nature and is therefore less prone to accumulation. Because cell membranes carry a negative charge, cationic NPs are more likely to bind and penetrate skin cells [60]. Nevertheless, there are positively charged sites on the surface of skin cells where even highly anionic NPs (-43 mV) can bind and penetrate the cell [61]. Interestingly, studies have shown that drug delivery to the skin using anionic NPs is often more efficient than that of cationic NPs [62]. Consequently, the precise role of the electrostatic potential of NPs in influencing drug binding, penetration, and release remains unclear and requires further investigation.

The polydispersity index (PDI) is another key property of NPs, ranging from 0 to 1, that reflects size uniformity. Values closer to 0 indicate a more uniform sample [63]. In this study, the PDI of ZnSe-*Uf* in our study was 0.31 on day 0 and 0.33 on day 90 (P > 0.05). A PDI below 0.05 is regarded as excellent, whereas values exceeding 0.7 suggest NPs are unsuitable for further investigations [63]. A low PDI (typically less than 0.3) signifies a narrow size distribution, which can enhance the stability of nanoparticle suspensions. This uniformity is critical in applications such as drug delivery, where consistent particle size can influence drug release profiles and bioavailability [64,65]. Studies have shown that silver nanoparticles with varying PDIs demonstrated different antibacterial activities, indicating that size uniformity can affect their efficacy against pathogens [66]. Similarly, nanoparticles with optimized PDIs have been associated with enhanced skin penetration for topical applications [67]. While a low PDI is generally desirable, it may not always correlate with optimal performance for all applications. Some formulations may benefit from a controlled range of sizes to achieve specific therapeutic effects or targeting capabilities [67,68]. ZnSe-*Uf* nanoparticles with a PDI 0.31–033 indicate moderate polydispersity. In contrast, many lipid-based nanoparticles are recommended to have PDIs below 0.3 for optimal performance [69,70]. This difference suggests that while ZnSe-*Uf* nanoparticles may still function effectively, they could face challenges in applications requiring high precision and consistency.

Numerous studies have highlighted the anti-parasitic properties of *Ulva* species [71,72]. Sulfated polysaccharides, particularly ulvan, are identified as the primary active compounds in these species, comprising 9% to 36% of their dry weight [73]. In our research, we analyzed the hydroalcoholic extract of *Ulva fasciata*, utilizing 80% ethanol as the extraction solvent. This solvent is known to efficiently isolate key active compounds, including polysaccharides [74]. The highest polysaccharide yield from *Ulva* species typically occurs between October and February [75], which is why we collected our samples during this timeframe.

Determining the precise ratio of NPs and the substance combined with them is essential for achieving optimal anti-leishmanial activity. Despite its importance, this aspect is often overlooked in many studies. Typically, the ratio of NPs to the combined substances ranges from 1:1 (w/w) to 1:10 (w/w), depending on the properties of both components [76–79]. In our research, a ratio of 1:1 (w/w) was considered for ZnSe NPs and *Ulva fasciata* extract. The optimization of the ratio of NPs to *Ulva fasciata* extract was achieved by conducting the experiments with varying variable values of pH [28,29], temperature [80], and reaction time [31], as well as different concentrations of the extract while keeping the against a constant concentration of ZnSe NPs [80]. However, further optimization of this ratio is recommended to enhance anti-leishmanial efficacy in future studies [81].

FTIR analysis identified seven key bonds within the main wavenumber range of 1,500 cm^-1^ to 4,000 cm^-1^, where the C-H group was dominant and attributed to polysaccharides, proteins, and lipids [36–39]. Moreover, the C-H group was also dominant in the fingerprint region (500 cm^-1^ to 1500 cm^-1^), corresponding to aromatic compounds [82]. In our study, peaks at 2936 cm^-1^ and 2881 cm^-1^ were closely resembled the peak with a shoulder at 2926 cm^-1^ reported by Sivakumar et al. [83]. In both cases, the first peak was shorter than the second. Following the second peak, the graph in both cases showed an upward trend, culminating in two additional peaks at 2368 cm^-1^ with a shoulder in our study and at 2358 cm^-1^ and 2343 cm^-1^ in Sivakumar et al.’s study [83]. Notably, the first peak in this later region was higher than the second in both investigations. Differences in wavenumbers between the two studies display that *Ulva fasciata* extract interacted with ZnSe NPs in our study, leading to shifts in wavenumber positions.

Compared to Cu NPs, with LC_50_ of 116.8 µg/mL and an SI of 11.34 [84], our study found ZnSe NPs to be significantly more toxic to amastigotes, with an LC_50_ of 2.29 µg/mL and an SI of 3.88. Similarly, Se NPs in another study demonstrated strong anti-leishmanial activity on amastigotes, showing an LC_50_ of 4.4 µg/mL and an SI of 2.38. This suggests that the presence of Se NPs could contribute to the observed toxicity of ZnSe NPs [85]. However, a notable reduction in ZnSe NPs toxicity was observed when combined with *Ulva fasciata* extract (SI: 27.55 for ZnSe-*Uf* compared to 3.88 for ZnSe). Similarly, such a trend was also observed with the combination of Cu NPs and the MA drug in a previous study [84].

Various studies have reported differing outcomes regarding the use of NPs against *L*. *major*. In our research, ZnSe-*Uf* demonstrated an impact on amastigotes with an LC_50_ of 24.3 µg/mL and an SI of 27.55, whereas Zr-tioxolone (NZT_1_) exhibited a weaker effect (LC_50_: 126.33 µg/mL) on *L*. *major* along with high toxicity (SI: 2.44) [86]. Conversely, artemether-loaded nanostructured lipid carriers (ART-NLCs) showed strong and safe effects (LC_50_: 11.92 µg/mL; SI: 59.42) [87]. Some studies studied the effects of NPs on *L*. *major* promastigotes and amastigotes without considering their SI [88–91], limiting the ability to interpret or compare those findings due to insufficient data.

The findings of our study indicate that while MA demonstrated greater effectiveness compared to ZnSe-*Uf*, the LC_50_ and SI values of ZnSe-*Uf* highlighted its anti-leishmanial properties and lack of toxicity. Therefore, further research is recommended to evaluate the impact of ZnSe-*Uf* on CL using an animal model. Additionally, it is essential to identify the optimal concentration of ZnSe NPs and *Ulva fasciata* extract when applied in combination.

## Conclusion

The study demonstrates that the incorporation of *Ulva fasciata* extract with ZnSe nanoparticles results in a stable composite with distinct morphological and functional characteristics, exhibiting significant antipromastigote and antiamastigote activity, as evidenced by higher SI values compared to ZnSe alone. ZnSe-*Uf* showed that it does not have a tendency to accumulate and is stable at 25 °C. Nevertheless, the effectiveness of ZnSe-*Uf* on amastigotes was less than that of MA, suggesting a need for further optimization in therapeutic applications.
